# Filling the gaps in the characterization of the clinical management of COVID-19: 30-day hospital admission and fatality rates in a cohort of 118 150 cases diagnosed in outpatient settings in Spain

**DOI:** 10.1093/ije/dyaa190

**Published:** 2020-10-29

**Authors:** Daniel Prieto-Alhambra, Elisabet Balló, Ermengol Coma, Núria Mora, María Aragón, Albert Prats-Uribe, Francesc Fina, Mència Benítez, Carolina Guiriguet, Mireia Fàbregas, Manuel Medina-Peralta, Talita Duarte-Salles

**Affiliations:** 1 Fundació Institut Universitari per a la Recerca a l'Atenció Primària de Salut Jordi Gol i Gurina (IDIAPJGol), Barcelona, Spain; 2 Centre for Statistics in Medicine, NDORMS, University of Oxford; 3 Sistemes d’Informació dels Serveis d’Atenció Primària (SISAP), Institut Català de la Salut (ICS), Barcelona, Spain; 4 Equip d’Atenció Primària de Salt, Institut Català de la Salut, Girona, Spain; 5 Equip d’Atenció Primària Gòtic, Institut Català de la Salut, Barcelona, Spain

**Keywords:** COVID-19, epidemiology, coronavirus, fatality, hospital admission

## Abstract

**Background:**

Currently, there is a missing link in the natural history of COVID-19, from first (usually milder) symptoms to hospitalization and/or death. To fill in this gap, we characterized COVID-19 patients at the time at which they were diagnosed in outpatient settings and estimated 30-day hospital admission and fatality rates.

**Methods:**

This was a population-based cohort study.

Data were obtained from Information System for Research in Primary Care (SIDIAP)—a primary-care records database covering >6 million people (>80% of the population of Catalonia), linked to COVID-19 reverse transcriptase polymerase chain reaction (RT-PCR) tests and hospital emergency, inpatient and mortality registers. We included all patients in the database who were ≥15 years old and diagnosed with COVID-19 in outpatient settings between 15 March and 24 April 2020 (10 April for outcome studies). Baseline characteristics included socio-demographics, co-morbidity and previous drug use at the time of diagnosis, and polymerase chain reaction (PCR) testing and results.

Study outcomes included 30-day hospitalization for COVID-19 and all-cause fatality.

**Results:**

We identified 118 150 and 95 467 COVID-19 patients for characterization and outcome studies, respectively. Most were women (58.7%) and young-to-middle-aged (e.g. 21.1% were 45–54 years old). Of the 44 575 who were tested with PCR, 32 723 (73.4%) tested positive. In the month after diagnosis, 14.8% (14.6–15.0) were hospitalized, with a greater proportion of men and older people, peaking at age 75–84 years. Thirty-day fatality was 3.5% (95% confidence interval: 3.4% to 3.6%), higher in men, increasing with age and highest in those residing in nursing homes [24.5% (23.4% to 25.6%)].

**Conclusion:**

COVID-19 infections were widespread in the community, including all age–sex strata. However, severe forms of the disease clustered in older men and nursing-home residents. Although initially managed in outpatient settings, 15% of cases required hospitalization and 4% died within a month of first symptoms. These data are instrumental for designing deconfinement strategies and will inform healthcare planning and hospital-bed allocation in current and future COVID-19 outbreaks.

**Key Messages**

- COVID-19 infections are spread in the community and are in fact more common amongst women in their middle ages. Almost one in five cases is seen in rural areas, with widespread distribution in all socio-economic strata.
- Severe forms of the disease, resulting in hospital admission or death, are more common in elderly men and nursing-home residents.
- Almost 15% of patients diagnosed with COVID-19 in outpatient settings need a hospital admission in the following month, with an overall 30-day fatality of 4%.

## Introduction

COVID-19 started as an outbreak in Wuhan, China, in December 2019 and rapidly developed into a global pandemic, causing a substantial morbidity and fatality burden, and straining healthcare systems worldwide.^1^ In Europe, it was first reported in late January 2020. The first case in Spain was reported a month later, although one study has suggested that community transmission were already occurring by then.^2^ By 26 July, Spain had reported the eighth-highest death toll of COVID-19 in the world.

As most COVID-19 patients present influenza-like symptoms, including fever, dry cough, fatigue and sore throat,^3^ they are eligible for outpatient or primary-care management in the first instance. However, studies to date have focused on the characteristics and prognosis of hospitalized^4^ or intensive-care^5^ COVID-19 patients, skewing current estimates of the morbi-mortality of COVID-19 globally. It is essential to characterize patients from their first diagnosis to achieve a more complete understanding of the prognosis of this disease.

Universal healthcare systems, such as those in Spain and the UK, rely on general practitioners to act as gatekeepers, with all patients seen in primary care before admission to hospital.^6^ Primary-care electronic health records from such healthcare systems, when linked with hospital emergency and inpatient and mortality registers, offer a unique opportunity to fully characterize the natural history of COVID-19.

We used outpatient and inpatient electronic medical records for a large number of COVID-19 cases, linked with reverse transcriptase polymerase chain reaction (RT-PCR) data and mortality registers, to describe the natural history of COVID-19 in Spain. We characterized COVID-19 patients’ socio-demographics, co-morbidities and medicines used at the time of diagnosis. We then estimated the need for hospital admissions and all-cause fatality associated with COVID-19 in the month following from first symptoms and outpatient diagnosis.

## Methods

### Study design and data sources

We performed a cohort study with prospectively collected data from the Information System for Research in Primary Care (SIDIAP; www.sidiap.org) in Catalonia, Spain.^7^ SIDIAP contains anonymized primary-care electronic health records for >6 million people, covering a representative >80% of the Catalan population since 2006. It includes high-quality, validated diagnoses [International Classification for Diseases, 10th revision, Clinical Modification (ICD-10-CM)], medicine prescriptions, laboratory tests, and lifestyle and socio-demographic information.^8^^,^^9^ For this study, SIDIAP was also linked to the region-wide population-based hospital and outpatient emergency register,^10^ the bespoke central database of RT-PCR COVID-19 tests and the regional mortality registry.

### Study participants and follow-up

We included all individuals aged ≥15 years with COVID-19 identified by a positive RT-PCR test for severe acute respiratory syndrome coronavirus 2 (SARS-CoV-2) and/or a clinical diagnosis (ICD-10-CM codes B34.2, B97.21, B97.29, J12.81) recorded in primary care from 15 March 2020 to 24 April 2020. We excluded individuals who were hospitalized with COVID-19 before their index date (prevalent cases). Patients with a prevalent diagnosis of pneumonia and/or a hospital admission for respiratory symptoms subsequently diagnosed with COVID-19 were also excluded to avoid misclassification. For outcome analysis (hospital admission or fatality), only those with an index date before 10 April 2020 were included to guarantee at least 14 days of follow-up from the index date.

Participants were followed from the earliest of a first positive RT-PCR test or clinical diagnosis (index date) until death or the end of the study period (24 April 2020). Repeat RT-PCR testing was dismissed.

### Baseline characteristics and co-morbidities

Socio-demographics were assessed at the index date: sex, age (in years), place of residence (community, nursing home), rurality (rural, urban) and nationality (Spain, other). Rural areas were defined as areas with <10 000 inhabitants and a population density of <150 inhabitants/km^2^. We assessed socio-economic status using the validated MEDEA deprivation index, which has previously been linked to SIDIAP^11^ and is calculated by the census tract level in urban areas, categorized in quartiles, where the first and fourth quartiles are the least and most deprived areas, respectively. Rural areas were categorized separately.

We defined pre-existing co-morbidities as the presence of a diagnosis code recorded at any time before the index date and still active at COVID-19 diagnosis for a pre-specified list of conditions based on previous literature.^3–5^ Lists of ICD-10-CM codes for each of these conditions are provided in Supplementary Table 1, available as Supplementary data at *IJE* online.

We characterized use of long-term medicines based on primary-care prescriptions active at the time of diagnosis (index date). A pre-specified list of medicines was created based on the same previous literature^3–5^ and identified using Anatomical Therapeutic Chemical Classification System codes (Supplementary Table 2, available as Supplementary data at *IJE* online).

### Outcomes

Two outcomes were studied, both 30 days after index date: COVID-19-related hospital admission and all-cause death. Hospitalizations were ascertained from linked hospital data covering emergency rooms and inpatient administrative data for the whole of Catalonia. COVID-19-related admissions were identified using a bespoke list of ICD-10-CM diagnostic codes recorded in hospital-discharge data (Supplementary Table 3, available as Supplementary data at *IJE* online). Date of death was obtained from linked regional mortality data.

### Statistical methods

For descriptive analyses, the median (inter-quartile range) or number (%) is reported for each patient characteristic. The cumulative number (%) of hospital admissions and fatality were obtained from the data. Thirty-day probabilities were obtained from Kaplan–Meier estimates, stratified by sex, age (10-year bands), nursing-home-residence status and RT-PCR result, where available. The Kaplan–Meier estimates and log-rank *p*-values are shown for both study outcomes, stratified by sex and age group. All analyses were conducted using R version 3.5.1.

### Ethics approval

This study was approved by the Clinical Research Ethics Committee of the IDIAPJGol (project code: 20/070-PCV). There was no patient or public involvement in this study.

## Results

We identified 118 150 cases of COVID-19 that were eligible for descriptive analyses, of whom 44 175 (37.7%) received an RT-PCR test. Test results were received within a median (inter-quartile range) of 3.9 (7.4) days and were mostly positive (32 723 cases, 73.4% of test), with 10 649 (23.4%) negative and 1202 (2.7%) inconclusive tests.

**Figure 1:**
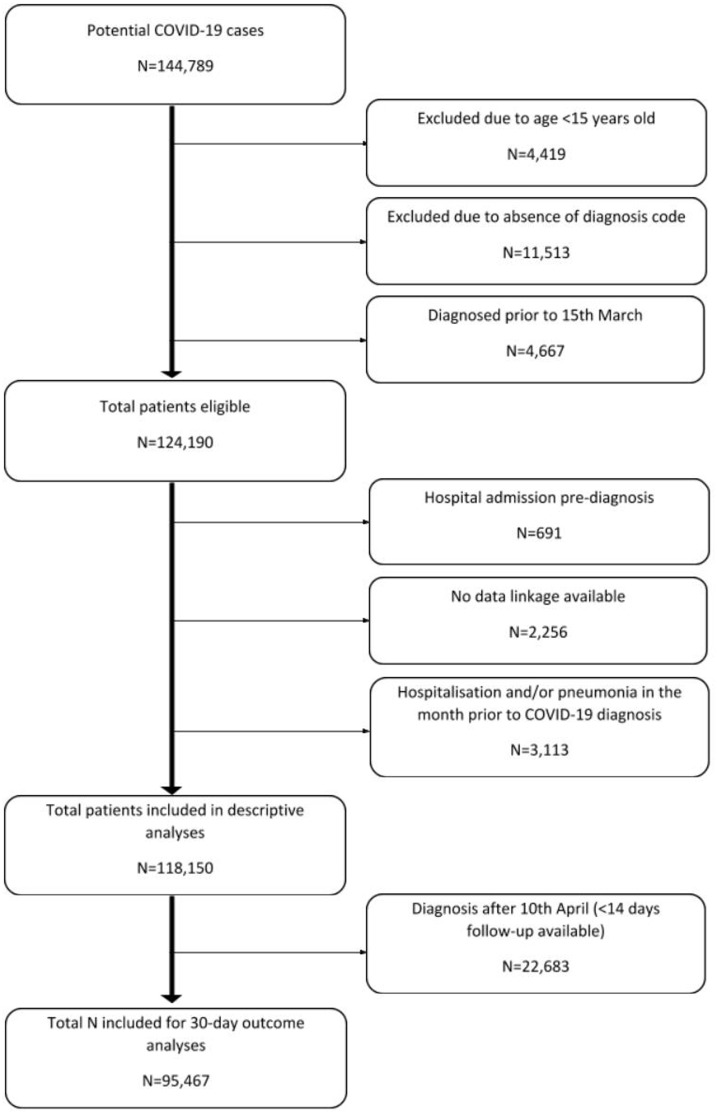
Population flow chart, showing the selection of patients for inclusion in each analysis

Patients with 14 or more days of follow-up (95 467/118 150; 80.8%) were included in the 30-day outcome analysis. Figure 1 shows the selection of patients for inclusion in each analysis.

Table 1 summarizes the included patients’ baseline characteristics when diagnosed with COVID-19. They were predominantly women (58.7%) who were middle-aged (21.1% 35–44, 21.1% 45–54 and 15.4% 55–64 years old); 21.2% (25 012) of infected people lived in the most deprived urban areas and an additional 17.6% (20 757) in rural areas. A substantial 10 452 patients (8.9%) lived in nursing homes and 587 (0.5% of total) women were pregnant when diagnosed with COVID-19.

**Table 1:** Baseline characteristics at the time of COVID-19 outpatient diagnosis^a^

Variable	Value	*N*	%
**Socio-demographics**			
Age	15–24 years old	6849	5.80%
Age	25–34 years old	15 949	13.50%
Age	35–44 years old	24 982	21.14%
Age	45–54 years old	24 949	21.12%
Age	55–64 years old	18 222	15.42%
Age	65–74 years old	9313	7.88%
Age	75–84 years old	8271	7.00%
Age	≥85 years old	9615	8.14%
Sex	Women	69 319	58.67%
Nationality	Spain	104 352	88.32%
Nationality	Other	13 798	11.68%
Socio-economic status	Urban—Quartile 1 (least deprived)	30 014	25.40%
Socio-economic status	Urban—Quartile 2	17 704	14.98%
Socio-economic status	Urban—Quartile 3	21 030	17.80%
Socio-economic status	Urban—Quartile 4 (most deprived)	25 012	21.17%
Socio-economic status	Rural	20 757	17.57%
Socio-economic status	Missing	3633	3.07%
Residence	Nursing home	10 452	8.85%
Pregnancy	Yes	587	0.50%
**Co-morbidities**			
Atrial fibrillation		4267	3.61%
Osteoarthritis		17 635	14.93%
Asthma		8025	6.79%
Ischaemic heart disease		3442	2.91%
Dementia		5303	4.49%
Diabetes mellitus		11 042	9.35%
Liver disease		5308	4.49%
Hypertension		27 789	23.52%
Heart failure		2687	2.27%
Cerebrovascular disease		2622	2.22%
Chronic obstructive pulmonary disease		3539	3.00%
Chronic kidney disease		5988	5.07%
Cancer (all except non-melanoma skin cancer)		8563	7.25%
Obesity		23 109	19.56%
HIV infection		300	0.25%
Hepatitis B		301	0.25%
Hepatitis C		710	0.60%
**Long-term medicine/s use**			
Analgesics		25 436	21.53%
Sedatives/hypnotics		19 684	16.66%
Anti-coagulants		15 202	12.87%
Antidepressants		18 860	15.96%
Anti-epileptics		7410	6.27%
Anti-psychotics		7525	6.37%
Proton-pump inhibitors/anti-acids		21 708	18.37%
Systemic corticosteroids		2539	2.15%
Oral anti-diabetics		7923	6.71%
Insulin		3323	2.81%
Lipid-modifying agents		14 398	12.19%
Chronic obstructive pulmonary disease/asthma inhalers		12 297	10.41%
Alpha-blockers		649	0.55%
Beta-blockers		8062	6.82%
Calcium channel blockers		6513	5.51%
Diuretics		9392	7.95%
ACEi/ARBs		13 448	11.38%
Combination anti-hypertensives		6209	5,26%

a Data are for patients with a positive result on a reverse transcriptase polymerase chain reaction (RT-PCR) test for severe acute respiratory syndrome coronavirus 2 (SARS-CoV-2) and/or a clinical diagnosis recorded in primary care from 15 March to 24 April 2020.

ACEi/ARBs, angiotensin-converting enzyme inhibitors/angiotensin receptor blockers.

The most common co-morbidities were hypertension (23.5%), obesity (19.6%) and osteoarthritis (14.9%). The least common co-morbidities were chronic viral hepatitis (0.6% type C, 0.3% type B) and HIV (0.3%). The 10 most commonly used long-term therapies at COVID-19 diagnosis were analgesics (21.5%), proton-pump inhibitors/anti-acids (18.4%), sedatives/hypnotics (16.7%), antidepressants (16.0%), anti-thrombotics (12.9%), lipid-modifying agents (12.2%), angiotensin-converting enzyme inhibitors/angiotensin receptor blockers (ACEi/ARBs) (11.4%), chronic obstructive pulmonary disease/asthma inhalers (10.4%), diuretics (8.4%) and beta-blockers (6.8%).

**Figure 2:**
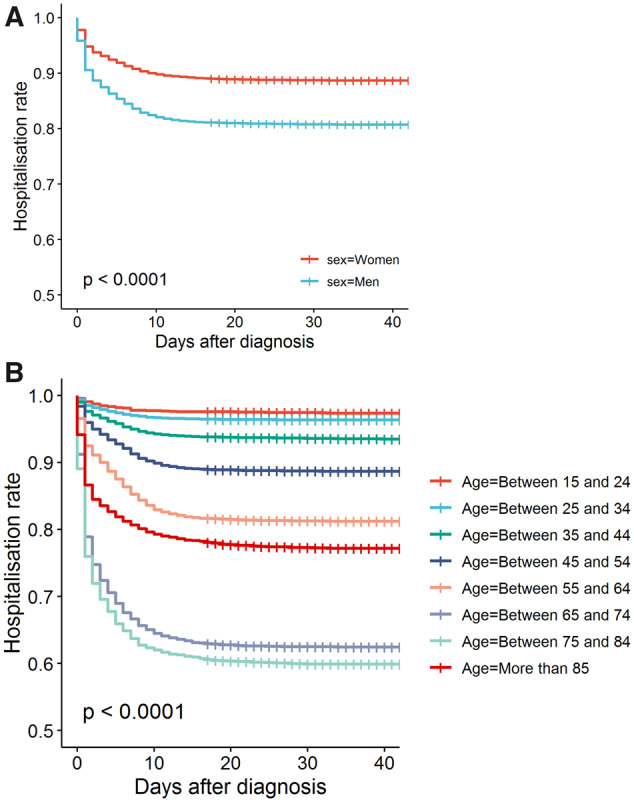
(A) Kaplan–Meier estimates of COVID-19-related hospital admission after COVID-19 diagnosis, stratified by sex. (B) Kaplan–Meier estimates of COVID-19-related hospital admission after COVID-19 diagnosis, stratified by age in 10-year bands.

In the month after diagnosis, 14 141 of the 95 467 patients were hospitalized for complications of COVID-19, equivalent to a cumulative incidence of 14.8% (14.6% to 15.0%). The incidence was higher among men [19.4% (19.0% to 19.8%)] than among women (*p* < 0.0001) (Figure 2A) and peaked at age 75–84 years old, with a striking 40.5% (39.3% to 41.7%) admitted within a month of diagnosis (*p* < 0.0001) (Figure 2B).

**Figure 3:**
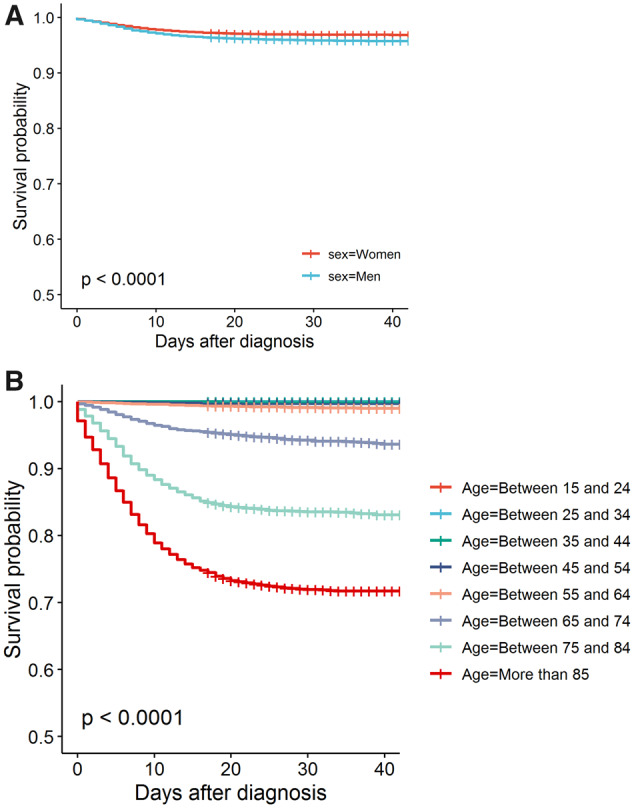
(A) Kaplan–Meier estimates of all-fatality after COVID-19 diagnosis, stratified by sex. (B) Kaplan–Meier estimates of all-cause fatality after COVID-19 diagnosis, stratified by age in 10-year bands.

We observed 3368 deaths among 95 467 patients, a 30-day fatality of 3.5% (3.4% to 3.6%). Figure 3A shows that all-cause fatality was higher among men [4.1% (4.0% to 4.3%)] than among women [3.1% (2.9% to 3.2%)] (*p* < 0.0001). Fatality increased monotonically with age and more steeply in the elderly, with all-cause fatality reaching 5.8% (5.3% to 6.3%) in those aged 65–74 years after 30 days, 16.3% (15.4% to 17.3%) in those aged 75–84 years and 27.4% (26.3% to 28.6%) in those aged ≥85 years (*p* < 0.0001) (Figure 3B).

Of the patients who had received an RT-PCR test, those with a positive result had a much higher cumulative incidence of hospital admissions and all-cause fatality than those with a negative result [hospital admissions: 43.1% (42.5% to 43.7%) in RT-PCR+ vs 15.6% (14.8% to 16.3%) in RT-PCR–; all-cause fatality: 5.7% (5.4% to 6.0%) in RT-PCR+ vs 0.5% (0.3% to 0.6%) in RT-PCR–].

Outcomes were dramatically different for those residing in nursing homes. Although their hospital-admissions rate was only 1.5% points higher than the whole population’s average, at 16.1% (15.1% to 17.0%), their 30-day all-cause fatality was >6-fold higher, at 24.5% (23.4% to 25.6%).

## Discussion

### Summary of key findings

To our knowledge, this is the first study of the characteristics and key health outcomes of COVID-19 among patients followed from first diagnosis in outpatient and primary-care settings. The existence of a universal tax-funded healthcare system with fully implemented electronic medical records covering out- and inpatient settings, linked to region-wide PCR testing and mortality registers, allowed us to fill a gap in the knowledge of the natural history of COVID-19.

We identified 118 150 people diagnosed with COVID-19 between 15 March and 24 April 2020 in Catalonia, Spain. Most of these people were managed in primary-care and outpatient settings, and only <40% were formally tested for the SARS-CoV-2 virus. This diagnosis figure was more than double the official figure of 48 916 cases in Catalonia by 30 April 2020, based on RT-PCR-confirmed cases (https://covid19.isciii.es/). A much higher proportion of the population may therefore have been affected, with different levels of disease severity, and community transmission may have been much wider than previously estimated from official figures.

### Findings in context

COVID-19 was spread throughout the studied community, with almost 60% of cases in women and most cases seen in young to middle-aged adults aged 35–65 years old. Infections were seen in all socio-economic strata in urban environments, and almost one in five patients lived in rural areas. However, more severe forms of COVID-19 disease were clustered in men and elderly people, who accounted for most of the hospital admissions and deaths. This result is in line with those from studies of hospitalized COVID-19 populations in the US,^12^ China^4^ and Europe,^13^ and suggests that age and sex are key risk factors for poor outcomes among those infected with COVID-19.

Chronic non-communicable diseases were common among patients with COVID-19. Probably related to this, >1 in 10 of the affected patients used medicines such as analgesics, proton-pump inhibitors/anti-acids, statins and anti-hypertensives such as ACEi/ARBs. Similar figures have been published elsewhere. Early reports from inpatient cases in China reported that 7.4% of COVID-19 patients had diabetes and 15% hypertension.^3^ More recently, an international study of 6806 patients hospitalized with COVID-19 in the US and South Korea^14^ found geographical variation in the prevalence of chronic co-morbidities. For example, 17.9% of COVID-19 patients in South Korea and 43.3% of US veterans with COVID-19 had diabetes, whereas 21.8% and 69.7% of these patients had hypertension. Patients in our data set who were treated as outpatients appeared somewhat healthier than the US and South Korean inpatient cases, but had a higher prevalence of co-morbidities than those reported from China: 9.75% had diabetes and 24.26% had hypertension. Our data and those obtained from previous studies suggest that the profile of patients with COVID-19 varies geographically, with no consistent differences seen between outpatient and inpatient cases.

Almost 15% of the patients with COVID-19 in our study were admitted to hospital in the month after their diagnosis in primary-care/outpatient settings. A preprint of a recent study compared hospital admissions for influenza and COVID-19, finding that COVID-19-admitted patients were generally younger and healthier than those admitted for influenza.^14^ Even though our sample included milder forms of COVID-19, 30-day fatality was still high at 4% on average and almost 5% in men. For comparison, estimated fatality rates for seasonal influenza ranged between 0.2% and 0.35% for the previous 13 influenza seasons in Catalonia.^15^ Previous vaccination^16^ and different age distributions are obvious contributors to influenza’s 10-fold lower fatality rate.

Fatality was much higher (about one in four) in older populations and nursing-home residents than the full study sample. A study of 101 COVID-19 residents of long-term care facilities in the US reported a fatality rate of 33.7%.^17^ The spread and severity of COVID-19 shown in this population reflect the recognized vulnerability of nursing-home residents to respiratory infections and airborne epidemics, including previous coronavirus^18^ and influenza outbreaks and pneumonia.^19^^,^^20^

COVID-19 testing has been limited in primary-care settings, even among symptomatic patients. Less than 40% of the identified cases in our sample had received an RT-PCR test, probably representing the more severe cases. A positive test result was a clear marker of prognosis, with 43% subsequently hospitalized and >6% dying within the next 30 days, compared with 15% and <1%, respectively, among clinically diagnosed cases who tested negative for COVID-19. Despite WHO recommendations, there are currently huge disparities across countries in the implementation of testing.^21^ Our study demonstrates the value of testing for planning healthcare-resource allocation, in addition to informing public-health decision-making. With the emergence of effective anti-COVID-19 therapies in the coming months, further testing will probably be needed for differential diagnosis.^22^^,^^23^

### Strengths and weaknesses of the study

Our study has limitations. Our large, routinely collected data set may have included misclassified cases, as other influenza-like illnesses or respiratory conditions could have been diagnosed as COVID-19 in the context of the current pandemic. In the subsample of patients with RT-PCR data available, almost three in four cases were positive, suggesting that the positive predictive value of outpatient diagnosis in our context approached 75%. As the other one in four patients had better health outcomes (fewer hospitalizations and lower fatality), it is likely that any misclassification led to underestimated risks of complications, admissions and fatality related to COVID-19 infection in our study.

Although we included milder forms of COVID-19 than previous characterization studies by including primary-care diagnoses, our sample will have missed most asymptomatic cases, as testing was not widespread in those without symptoms. The sample also likely missed many of those with mild symptoms, as, following official advice to stay home and self-isolate to avoid contact and spread in healthcare facilities, they may not have ever come into contact with their primary-care practice or a hospital to receive an official diagnosis.

Our study’s strengths lie in our comprehensive data set. The use of routinely collected data allowed a large sample size. SIDIAP is a well-validated data source^9^ that has been used in many previous studies.^24^ By using primary-care records linked to comprehensive region-wide hospital, mortality and testing registers obtained from a universal tax-funded healthcare system, we were able to completely characterize the natural history of COVID-19 infection from symptom onset, while avoiding the recall bias and loss to follow-up typical of cohort studies.

Including COVID-19 cases treated exclusively in outpatient settings allowed the study of subpopulations that are less likely to be admitted to hospital and are known to be more susceptible to infection. For instance, we identified almost 11 000 nursing-home residents and >9000 people with a history of cancer who were diagnosed with COVID-19, and almost 600 women diagnosed with COVID-19 during pregnancy. Our study data set thus included the largest numbers of COVID-19 cases among these populations yet recorded. For comparison, previous studies of COVID-19 patients with prevalent cancer have included <30 participants.^25^^,^^26^ This data set will be an invaluable resource for studying the effects of SARS-CoV-2 in these populations. Finally, linkage to test data and to comprehensive region-wide hospital and mortality registers allowed us to track COVID-19 infections through a universal healthcare system, whilst avoiding recall bias and loss to follow-up typical of cohort studies.

## Conclusion

This is the first study to date on the characteristics, hospital admissions and fatality associated with COVID-19 disease diagnosed in outpatient settings. COVID-19 is often diagnosed and initially managed in outpatient clinics, with limited testing leading to underreported cases in official figures and overestimated fatality. Notwithstanding this, COVID-19 in our wide sample led to hospitalization in 15% of diagnosed patients and a 30-day fatality of 4%. Our data suggest that, although COVID-19 infection has spread throughout the Catalonia, Spain community, most often in young and middle-aged women, severe forms of the disease and all-cause fatality cluster in older men and among nursing-home residents. This information is of key relevance for healthcare professionals, public-health authorities and commissioners, now and in future COVID-19 outbreaks.

## Supplementary data

Supplementary data are available at *IJE* online.

## Author contributions

All authors contributed to the design of the study and interpretation of the results, and reviewed the manuscript. E.C. and N.M. had access to the data, performed the statistical analysis and acted as guarantors. D.P.A., E.C., N.M., A.P.-U. and T.D.S. wrote the first draft of the manuscript. All authors critically revised the manuscript. The corresponding author attests that all listed authors meet authorship criteria and that no others meeting the criteria have been omitted.

## Funding

This project is funded by the Health Department from the Generalitat de Catalunya with a grant for research projects on SARS-CoV-2 and COVID-19 disease organized by the Direcció General de Recerca i Innovació en Salut. D.P.A. is funded through a National Institute for Health Research (NIHR) Senior Research Fellowship (grant number SRF-2018–11-ST2-004). The views expressed in this publication are those of the author(s) and not necessarily those of the NHS, the National Institute for Health Research or the Department of Health. A.P.-U. is supported by Fundacion Alfonso Martin Escudero and the Medical Research Council (grant numbers MR/K501256/1, MR/N013468/1). The University of Oxford received a grant related to this work from the Bill & Melinda Gates Foundation (Investment ID INV-016201).

## Supplementary Material

dyaa190_Supplementary_Data

## References

[1] COVID-19 Map—Johns Hopkins Coronavirus Resource Center. https://coronavirus.jhu.edu/map.html.

[2] Díez-Fuertes F, Iglesias-Caballero M, Monzón S, et al Phylodynamics of SARS-CoV-2 transmission in Spain. bioRxiv. doi: 10.1101/2020.04.20.050039, 20 April 2020, preprint: not peer reviewed.

[3] Guan W-J, Ni Z-y, Hu Y et al Clinical characteristics of coronavirus disease 2019 in China. N Engl J Med 2020;382:1708–20. 32109013 10.1056/NEJMoa2002032 PMC7092819

[4] Zhou F, Yu T, Du R et al Clinical course and risk factors for mortality of adult inpatients with COVID-19 in Wuhan, China: a retrospective cohort study. Lancet 2020;395:1054–62. 32171076 10.1016/S0140-6736(20)30566-3 PMC7270627

[5] Grasselli G, Zangrillo A, Zanella A et al Baseline characteristics and outcomes of 1591 patients infected with SARS-CoV-2 admitted to ICUs of the Lombardy Region, Italy. JAMA 2020;323:1574–81. 32250385 10.1001/jama.2020.5394 PMC7136855

[6] Gérvas JUAN, Ferna MP, Starfield BH. Primary care, financing and gatekeeping in Western Europe. Fam Pract 1994;11:307–17. 7843523 10.1093/fampra/11.3.307

[7] García-Gil MD M, Hermosilla E, Prieto-Alhambra D et al Construction and validation of a scoring system for the selection of high-quality data in a Spanish population primary care database (SIDIAP). Inform Prim Care 2011;19:135–45. 22688222 10.14236/jhi.v19i3.806

[8] Ponjoan A, Garre-Olmo J, Blanch J et al How well can electronic health records from primary care identify Alzheimer's disease cases? Clin Epidemiol 2019;11:509–18. 31456649 10.2147/CLEP.S206770 PMC6620769

[9] Recalde M, Manzano-Salgado CB, Diaz Y et al Validation of cancer diagnoses in electronic health records: results from the information system for research in primary care (SIDIAP) In Northeast Spain. Clin Epidemiol 2019;11:1015–24. 31819655 10.2147/CLEP.S225568 PMC6899079

[10] Divisió d'Anàlisi de la Demanda i d'Activitat. Informe de L'activitat Notificada al Registre Del Conjunt Mínim Bàsic de Dades D’urgències (CMBD-UR), Any 2015. Barcelona: Departament de Salut, 2016.

[11] Garcia-Gil M, Elorza JM, Banque M et al Linking of primary care records to census data to study the association between socioeconomic status and cancer incidence in Southern Europe: a nation-wide ecological study. PLoS One 2014;9:e109706. 25329578 10.1371/journal.pone.0109706 PMC4203762

[12] S G, L K, M W. Hospitalization rates and characteristics of patients hospitalized with laboratory-confirmed coronavirus disease 2019—COVID-NET, 14 States, March 1–30, 2020. MMWR Morb Mortal Wkly Rep 2020;69:458–64. 32298251 10.15585/mmwr.mm6915e3 PMC7755063

[13] Docherty AB, Harrison EM, Green CA, et al Features of 16,749 hospitalised UK patients with COVID-19 using the ISARIC WHO Clinical Characterisation Protocol. medRxiv, doi: 10.1101/2020.04.23.20076042, 23 April 2020, preprint: not peer reviewed.

[14] Burn E, You SC, Sena A, et al Deep phenotyping of 34,128 patients hospitalised with COVID-19 and a comparison with 81,596 influenza patients in America, Europe and Asia: an international network study. medRxiv, doi: 10.1101/2020.04.22.20074336, 22 April 2020, preprint: not peer reviewed. PMC7538555 33024121

[15] Munoz MP, Soldevila N, Martinez A et al Influenza vaccine coverage, influenza-associated morbidity and all-cause mortality in Catalonia (Spain). Vaccine 2011;29:5047–52. 21620915 10.1016/j.vaccine.2011.04.067

[16] Campitelli MA, Rosella LC, Stukel TA, Kwong JC. Influenza vaccination and all-cause mortality in community-dwelling elderly in Ontario, Canada, a cohort study. Vaccine 2010;29:240–46. 21044667 10.1016/j.vaccine.2010.10.049

[17] McMichael TM, Currie DW, Clark S et al Epidemiology of Covid-19 in a long-term care facility in King County, Washington. N Engl J Med 2020;382:2005–11. 32220208 10.1056/NEJMoa2005412 PMC7121761

[18] Hand J, Rose EB, Salinas A et al Severe respiratory illness outbreak associated with human coronavirus NL63 in a long-term care facility. Emerg Infect Dis 2018;24:1964–66. 30226169 10.3201/eid2410.180862 PMC6154147

[19] Bosco E, Zullo AR, McConeghy KW et al Geographic variation in pneumonia and influenza in long-term care facilities: a national study. Clin Infect Dis 2020;ciaa081. 10.1093/cid/ciaa081 PMC7643743 31995171

[20] Childs A, Zullo AR, Joyce NR et al The burden of respiratory infections among older adults in long-term care: a systematic review. BMC Geriatr 2019;19:210. 31382895 10.1186/s12877-019-1236-6 PMC6683564

[21] Cohen J, Kupferschmidt K. Countries test tactics in ‘war’ against COVID-19. Science 2020;367:1287–88. 32193299 10.1126/science.367.6484.1287

[22] Ledford H. Hopes rise for coronavirus drug remdesivir. Nature 2020;10.1038/d41586-020-01295-8. 10.1038/d41586-020-01295-8 32350436

[23] Grein J, Ohmagari N, Shin D et al Compassionate use of Remdesivir for patients with severe Covid-19. N Engl J Med 2020;382:2327–36. 32275812 10.1056/NEJMoa2007016 PMC7169476

[24] Ramos R, Comas-Cufi M, Marti-Lluch R et al Statins for primary prevention of cardiovascular events and mortality in old and very old adults with and without type 2 diabetes: retrospective cohort study. BMJ 2018;362:k3359. 30185425 10.1136/bmj.k3359 PMC6123838

[25] Zhang L, Zhu F, Xie L et al Clinical characteristics of COVID-19-infected cancer patients: a retrospective case study in three hospitals within Wuhan, China. Ann Oncol 2020;31:894–901. 32224151 10.1016/j.annonc.2020.03.296 PMC7270947

[26] Liang W, Guan W, Chen R et al Cancer patients in SARS-CoV-2 infection: a nationwide analysis in China. Lancet Oncol 2020;21:335–37. 32066541 10.1016/S1470-2045(20)30096-6 PMC7159000

