# Supplementary material for: Filling the gaps in the characterization of the clinical management of COVID-19: 30-day hospital admission and fatality rates in a cohort of 118 150 cases diagnosed in outpatient settings in Spain
Source: Int J Epidemiol. 2020 Oct 29;49(6):1930–9. doi: 10.1093/ije/dyaa190 (PMC7665572; doi:10.1093/ije/dyaa190)
Supplement: dyaa190_Supplementary_Data [file dyaa190_supplementary_data.docx]

# Supplementary information

**Table S1.** Comorbidities of interest and related code lists

| **Comorbidity** | **ICD10-CM codes** |
| --- | --- |
| Pregnancy | O07-O11, O22, O24-O26, O30-O36, O40-O45, O47-O48, O88, O90, O94, Z32-Z34 |
| Hypertension | I10, I11.0, I11.9, I12.0, I12.9, I13.0, I13.10, I13.2, I15.0,I15.1,I15.2, I15.8,I15.9 |
| Type 1 Diabetes Mellitus | E10.10, E10.29, E10.311, E10.359, E10.39, E10.49, E10.59, E10.621, E10.65, E10.69, E10.8, E10.9 |
| Type 2 Diabetes Mellitus | E11.01, E11.21, E11.22, E11.29, E11.311, E11.39, E11.40, E11.43, E11.49, E11.51, E11.59, E11.610. E11.621, E11.638, E11.641, E11.649, E11.65, E11.69, E11.8, E11.9, E13.10, E13.29, E13.39, E13.49, E13.59, E13.641, E13.69, E13.8, E13.9 |
| Chronic obstructive pulmonary disease (COPD) | J43.0, J43.1, J43.2, J43.8, J43.9, J44, J44.0,J44.1,J44.9 |
| Asthma | J45, J45.20, J45.21, J45.22,  J45.40, J45.50, J45.901, J45.902, J45.998 |
| Ischaemic heart disease | I20, I20.0, I20.8, I20.9, I24.0, I24.1, I24.8, I24.9, I25, I25.10, I25.2, I25.41, 125.5, I25.6, I25.89, I25.9, I70.90, I21, I21.0, I21.01, I21.02,I21.09, I21.1, I21.11, I21.19, I21.2, I21.29, I21.3, I21.4, I22.0, I22.1, I22.2, I22.8, I22.9, I23.0, I23.1, I23.2, I23.3, I23.4, I23.5, I23.6, I23.8, |
| Cerebrovascular disease | G45.0, G45.1, G45.2, G45.8, G45.9, G46.0, G46.1, G46.2, G46.3, G46.4, G46.5, G46.6, G46.7, G46.9, I63.00, I63.10, I63.20, I63.30, I63.40, I63.50, I63.6, I63.8, I63.9, I67.82, I67.9, I69.30, I69.320 I69.398, I69.80,I69.998  I61.0, I61.1, I61.2, I61.3, I61.4, I61.5, I61.6, I61.8, I61.9, I61.10 |
| Heart failure | I50.1, I50.20, I50.30, I50.32, I50.9 |
| Atrial fibrillation | I48.0. I48.1, I48.2, I48.91 |
| Liver failure | K70.0, K70.10, K70.2, K70.30, K70.9, K73.0, K73.1, K73.2, K73.8, K73.9, K75.0, K75.2, K75.3, K75.4, K75.89, k75.9, K76.5, K76.7, K76.9 |
| Type B Hepatitis | B16.0, B16.1, B16.2, B16.9, |
| Type C Hepatitis | B17.10, B18.2 |
| Cancer (all except non-melanoma skin cancer) | C00-C97 (except C44), D00-D09 |
| Chronic Kidney Disease | I12.0, I13.0, N03.9, N18.1, N18.2, N18.4, N18.5, N18.6, N18.9, N19, N28.9, N99.0, P96.0, Z94.0 |
| Obesity | E66.01, E66.09, E66.1, E66.2, E66.8, E66.9 |
| HIV infection | Z21,B20, B97.35 |

**Table S2.** Long-term medications of interest and related code lists

| **Medicine/s or drug class** | **ATC codes** |
| --- | --- |
| Analgesics | M01AX%, N02AA%, N02AB%, N02AC%,N02AD%, N02AE%, N02AX%, N02BA%, N02BB%, N02BE%  M01AA%, M01AB%, M01AC%, M01AE%, M01AG%, M01AH%. |
| Sedatives | N05BA%, N05BB%, N05BE%, N05BX%, N05CD%, N05CF%, N05CM%, |
| Antithrombotics | B01AA%, B01AB%, B01AC%, B01AE%, B01AF%, B01AX%, |
| Antidepressants | N06AA%, N06AB%, N06AG%, N06AX%, |
| Antiepileptics | N03AA%, N03AB%, N03AC%, N03AD%, N03AE%, N03AF%, N03AG%, N03AX%, |
| Antipsychotics | N05AA%, N05AB%, N05AC%, N05AD%, N05AE%, N05AF%, N05AH%, N05AX%, N05AK%, N05AL% |
| Antiacids | A02AB%, A02AD%, A02AX%, A02BA%, A02BB%, A02BC%, A02BX%, |
| Systemic corticosteroids | H02AB%, H02BX91 |
| ACEi/ARBs | C09AA%, C09CA% |
| Calcium channel blockers | C08CA%, C08DA%, C08DB% |
| Thiazidic diuretics | C03AA%, C03BA% |
| Potassium sparent diuretics | C03DA%, C03DB% |
| Diuretics in combination | C03EA%, C03EB% |
| Beta blockers | C07AA%, C07AB%, C07AG% |
| Alpha blockers | C02CA% |
| Oral antidiabetic agents | A10BA%, A10BB%, A10BD%, A10BF%, A10BG%,A10BH%, A10BX%, A10BK%, A10BJ% |
| Insulin | A10AB%, A10AC%,A10AD%, A10AE% |
| Lipid modifying agents | C10AA%  C10AB%, C10AC%, C10AD%, C10AX%, C10BA%, C10BX%, |
| Inhalers | R03AA%, R03AC%, R03AK%, R03AL%, R03BA%, R03BB% |

**Table S3**. Code lists for hospital admissions attributable to COVID-19 disease or complications

| **ICD-10-CM** | **Description** |
| --- | --- |
| B34.2 | Coronavirus infection, unspecified |
| B97.29 | Other coronavirus as the cause of diseases classified elsewhere |
| B97.89 | Other viral agents as the cause of diseases classified elsewhere |
| B34.9 | Viral infection, unspecified |
| B97.21 | SARS-associated coronavirus as the cause of diseases classified elsewhere |
| J11% | Influenza due to unidentified influenza virus |
| J12% | Viral pneumonia, not elsewhere classified |
| J13% | Pneumonia due to Streptococcus pneumoniae |
| J14% | Pneumonia due to Hemophilus influenzae |
| J15% | Bacterial pneumonia, not elsewhere classified |
| J16% | Pneumonia due to other infectious organisms, not elsewhere classified |
| J17% | Pneumonia in diseases classified elsewhere |
| J18% | Pneumonia, unspecified organism |
| J20% | Acute bronchitis |
| J22% | Unspecified acute lower respiratory infection |
| J40% | Bronchitis, not specified as acute or chronic |
| J41% | Simple and mucopurulent chronic bronchitis |
| J42% | Unspecified chronic bronchitis |
| J43% | Emphysema |
| J44% | Other chronic obstructive pulmonary disease |
| J45% | Asthma |
| J47% | Bronchiectasis |
| J80% | Acute respiratory distress syndrome |
| J84% | Other interstitial pulmonary diseases |
| J96% | Respiratory failure, not elsewhere classified |
| J98.8 | Other specified respiratory disorders |
| J06.9 | Acute upper respiratory infection, unspecified |
| R50.9 | Fever, unspecified |
| R06.09 | Other forms of dyspnea |
| Z03.818 | Encounter for observation for suspected exposure to other biological agents ruled out |
| R06.00 | Dyspnea, unspecified |
| R05 | Cough |
| R53.1 | Weakness |
| R09.02 | Hypoxemia |
| R19.7 | Diarrhea, unspecified |
| I50.9 | Heart failure, unspecified |
| Z11.59 | Encounter for screening for other viral diseases |
| Z20.828 | Contact with and (suspected) exposure to other viral communicable diseases |
